# Lack of a *p21^waf1/cip^*-Dependent G1/S Checkpoint in Neural Stem and Progenitor Cells After DNA Damage In Vivo

**DOI:** 10.1002/stem.1010

**Published:** 2011-12-12

**Authors:** Telma Roque, Céline Haton, Olivier Etienne, Alexandra Chicheportiche, Laure Rousseau, Ludovic Martin, Marc-André Mouthon, François D Boussin

**Affiliations:** a CEA, DSV IRCM SCSR, Laboratoire de RadiopathologieUMR 967, Fontenay-aux-Roses, France; b INSERM, U967Fontenay-aux-Roses, France; c Univ Paris Diderot, Sorbonne Paris Cité, UMR 967Fontenay-aux-Roses, France; d Univ Paris-Sud, UMR 967Fontenay-aux-Roses, France

**Keywords:** *p21^waf1/cip^*, G1/S checkpoint, Neural stem cells, Ionizing radiation, DNA damage response, Cell cycle

## Abstract

The cyclin-dependent kinase inhibitor p21^waf1/cip^ mediates the p53-dependent G1/S checkpoint, which is generally considered to be a critical requirement to maintain genomic stability after DNA damage. We used staggered 5-ethynyl-2′deoxyuridine/5-bromo-2′-deoxyuridine double-labeling in vivo to investigate the cell cycle progression and the role of p21^waf1/cip^ in the DNA damage response of neural stem and progenitor cells (NSPCs) after exposure of the developing mouse cortex to ionizing radiation. We observed a radiation-induced p21-dependent apoptotic response in migrating postmitotic cortical cells. However, neural stem and progenitor cells (NSPCs) did not initiate a *p21^waf1/cip1^*-dependent G1/S block and continued to enter S-phase at a similar rate to the non-irradiated controls. The G1/S checkpoint is not involved in the mechanisms underlying the faithful transmission of the NSPC genome and/or the elimination of critically damaged cells. These processes typically involve intra-S and G2/M checkpoints that are rapidly activated after irradiation. p21 is normally repressed in neural cells during brain development except at the G1 to G0 transition. Lack of activation of a G1/S checkpoint and apoptosis of postmitotic migrating cells after DNA damage appear to depend on the expression of p21 in neural cells, since substantial cell-to-cell variations are found in the irradiated cortex. This suggests that repression of p21 during brain development prevents the induction of the G1/S checkpoint after DNA damage. Stem Cells *2012;30:537–547*

## INTRODUCTION

Faithful transmission of the genome after genotoxic stress during cell division depends on an appropriate DNA damage response (DDR), which includes the accurate detection and repair or elimination of damaged cells. This is a major challenge for stem and progenitor cells, which must preserve further self-renewal and differentiation capacities. The induction of cell cycle checkpoints is an essential component of DDR in cycling cells as it is thought to provide ample time for DNA repair or for the elimination of severely damaged and potentially hazardous cells. Three main cell checkpoints have been described to operate after double-strand breaks: the G1/S, intra-S, and G2/M checkpoints. p21^waf1/cip1^ (thereafter referred to as p21), a member of cip/kip family of cyclin-dependent kinase (CDK) inhibitors, is the key mediator of the p53-induced G1 checkpoint [1, 2] via its binding to and inactivation of G1-associated cyclin A- and cyclin E-containing cyclin/cdk complexes. p21 is thus usually considered to play a major role in DDR of cycling cells.

Radial glial cells function as neural stem cells in the developing mammalian cortex. Their progeny include neurons, astrocytes, oligodendrocytes, ependymocytes, and adult neural stem cells [3]. Radial glia span the entire developing cortex, but their nuclei are located within the ventricular zone (VZ), where they undergo interkinetic migration (INM), performing mitosis at the surface of ventricle and S-phase within the VZ [4–6]. Radial glial cells expand via symmetric divisions and generate through asymmetric divisions of projection neurons directly or indirectly via intermediate progenitors. Intermediate progenitors migrate in an overlying secondary structure, known as the subventricular zone (SVZ), where they undergo one or more divisions before differentiating into neurons. Immature neurons migrate through the intermediate zone (IZ) and take up laminar positions within the cortical plate (CP) [7]. The coordination of these different events requires a strict regulation of the cell cycle progression of neural stem and progenitor cells (NSPCs). The switch from expansion to differentiation in neural progenitor populations is controlled by the duration of the G1 phase [8]. p21, as well as another cyclin kinase inhibitor p27, has been linked to cell cycle exit during corticogenesis [9–12]. However, p21 expression is controlled by several transcription factors [9, 13–15] and limited to a short period at the G1 to G0 transition, whereas p27 alone is required for continued suppression of cycling activity in migrating and differentiating neurons [16].

NSPCs are highly prone to p53-dependent apoptosis after ionizing radiation exposure [17–19], but the activation of cell cycle checkpoints in these cells and the consequences of this for neurogenesis have not been fully investigated. We thus examined cell cycle progression in the developing cortex in *wt* and *p21^−/−^* mouse embryos irradiated in utero in this study. Our analysis revealed that ionizing radiation rapidly delays cell cycle progression and INM in NSPCs and induces apoptosis via intra-S and G2/M checkpoints that are activated independently of p21. We further show that p21 has a pro-apoptotic effect upon postmitotic migrating neurons in the VZ and SVZ of irradiated brains, but that irradiated NSPCs do not activate the p21-dependent G1/S checkpoint. Our finding of significant cell-to-cell variations in p21 nuclear expression in irradiated cortices further suggests that factors favoring the expansion of NSPCs may prevent radiation-induced p21 expression and thus block the G1/S checkpoint.

## MATERIALS AND METHODS

### Animals and Irradiation Procedure

Mouse experiments were carried out in compliance with all legal requirements for the experimental use of animals. In utero irradiations at 14.5 days of gestation (E14.5) were carried on female B6;129SV *p21^−/−^* and *wt* mice with a ^137^Cs source (IBL 637, CIS BIO International; 0.5, 1, 2, or 5 Gy, 0.61 Gy/minute). Sham-irradiated animals were treated in the same manner but not exposed to the source. Pregnant mice were sacrificed humanely at various time points after irradiation, and the embryos were removed and decapitated. Embryonic heads were fixed overnight at 4°C by immersion in 4% paraformaldehyde (PFA) or in 100% methanol at −20°C. Tissue was processed for paraffin embedding on a Tissu-Tek processor (VIP, Leica, Wetzlar, Germany). Coronal sections (5 μm) were then obtained and mounted onto glass slides for histologic analyses.

### Immunofluorescence Staining

Brain sections were deparaffinized, boiled for 10 minutes in citrate solution (pH 6), and then incubated in phosphate buffered saline (PBS) supplemented with 7.5% goat serum and 7.5% fetal bovine serum (FBS) for 1 hours at room temperature to block nonspecific antibody binding. Sections were then incubated with various primary monoclonal and polyclonal antibodies for 1 hours at room temperature. The primary antibodies used were anti-Pax6 (1:200, ref AB5409, Millipore, Billerica, MA, http://www.millipore.com), anticleaved caspase-3 (1:200, ref 9661, Cell Signaling Technology, Danvers, MA, http://www.cellsignal.com), anti-Tbr2 (1:100, ref ab23345, Abcam, Cambridge, U.K., http://www.abcam.com), anti-phospho-histone 3 (lys9/ser10) (1:100, ref 9711, Cell Signaling), and anti-phospho-Histone H2AX (1:200, ref 05-636, Millipore). After three washes, cells were incubated with secondary antibodies, either Alexa Fluor 594 or Alexa Fluor 488 immunoglobulin (1:400, Life Technologies, Carlsbad, CA, http://www.lifetech.com). Nuclear staining was achieved by incubation with 4′-6-diamidino-2-phenylindole (DAPI) to quantify apoptosis induction by the detection of pyknotic nuclei. Slides were mounted under Fluoromount (Southern Biotechnologies Associates, Birmingham, AL, http://www.southernbiotech.com), and brain tissues were examined under a fluorescence microscope (Olympus BX51) with a ×20 objective in three channels (appearing red, green, and blue in all figures) as separate files. These images were then stacked with Photoshop software (Adobe, San Jose, CA) and used for the enumeration of labeled cells.

### Terminal Deoxynucleotidyl Transferase Labeling

After paraffin removal, coronal sections were processed for terminal deoxynucleotidyl transferase labeling (TUNEL) histochemistry in accordance with the manufacturer's instructions (ref 1 684 795, Roche Diagnostics, Basel, Switzerland, http://www.roche-applied-science.com). Briefly, the cells were boiled in citrate solution (pH 6) and then incubated with the TUNEL reaction mixture that contained terminal deoxynucleotidyl transferase and fluorescein-dUTP for 1 hours at 37°C. After various washing steps, the labels incorporated at the damaged sites of the DNA were visualized by fluorescence microscopy.

### 5-Ethynyl-2′deoxyuridine and 5-Bromo-2′-deoxyuridine Administration

On embryonic day E14.5, pregnant mice received intraperitoneal (i.p.) injections of 0.1 mg 5-ethynyl-2′deoxyuridine (EdU; ref A10044, Life Technologies) at 1.5 hours before or just after irradiation, followed by 1 mg of 5-bromo-2′-deoxyuridine (BrdU) (ref B5002; Sigma, St. Louis, MO) at different time points after irradiation depending on the experiments. Embryos were then processed as described above. After paraffin removal, brain coronal sections were boiled in citrate solution pH 6 and permeabilized with 0.5% Triton X-100 in PBS for 20 minutes. EdU staining was performed in accordance with the manufacturer's protocol (Click-it EdU Alexa 488 imaging kit, ref C10083; Life Technologies). Briefly, the coronal sections were incubated with a Click-iT reaction cocktail containing Click-iT reaction buffer, CuSO_4_, Alexa Fluor 488 Azide, and reaction buffer additive for 30 minutes while protected from light. The sections were then blocked with 7.5% FBS and 7.5% goat serum in PBS for 1 hours and incubated with mouse anti-BrdU antibody (ref RPN202, GE Healthcare, Velizy, France, http://www.gehealthcare.com) at a 1:300 dilution in the blocking serum overnight at 4°C. After washing, the sections were incubated with Alexa Fluor 594-conjugated secondary antibody (1:400, Life Technologies) for 1 hours. After further washings, nuclear staining was achieved by incubation with DAPI for 5 minutes. The slides were then washed twice with PBS and coverslipped with Fluoromount mounting media (Southern Biotechnologies Associates). Images were captured under a fluorescence microscope (Olympus BX51).

### Immunohistochemistry

Brain sections were mounted onto glass slides, deparaffinized, and boiled for 10 minutes in citrate solution at pH 6. Endogenous peroxidase activity was blocked with 3% hydrogen peroxide for 10 minutes. The sections were then washed with PBS and blocked for 1 hours with 2.5% horse serum. Slides were incubated overnight at 4°C with mouse anti-p21 antibody (1:50, ref 556431, BD, Franklin Lakes, NJ, http://www.bd.com) or mouse anti-p53 antibody (1:50, ref 35165, Leica, Nanterre, France, http://www.leica.com) in the blocking serum and then with ImmPRESS reagent (Vector Laboratories, MP-7401, Burlingame, CA, http://www.vectorlabs.com) for 30 minutes. ImmPACT DAB (Vector Laboratories) was used as the chromogen and hematoxylin as the nuclear counterstain. Images were captured under a phase contrast microscope (Olympus BX51).

### Flow Cytometry

Pregnant mice (E14.5) were injected with BrdU (i.p., 1 mg) just after a 2 Gy irradiation. Embryonic cortices were microdissected at 4 hours post-irradiation (PI) and dissociated by incubation for 10 minutes with collagenase type I (100 U/ml, Life Technologies) and DNase (100 U/ml, Sigma). Isolated cells were subsequently fixed with 4% PFA. Immunostaining for BrdU was performed using an allophycocyanin–BrdU Flow Kit in accordance with manufacturer's protocol (ref 552598, BD). The cell DNA content was assessed via the 7-amino-actinomycin D staining pattern (ref B2261, Sigma). Cells were then analyzed on a FACSCalibur flow cytometer (BD), and the data were processed with FlowJo software (Ashland, OR, Version 7.2.5).

### Analysis of Cortical Tissue Samples

Cortical slices from a standard sector of the dorsomedial cerebral wall were analyzed as previously described [20]. This sector was 100 μm in its medial-lateral dimension and was divided into 18 bins of 10-μm height in its radial dimension. The sector was aligned such that the first bin was at the ventricle surface, with its long axis parallel to the ventricle border (Supporting Information Fig. S1A). The number of labeled (immunostaining for BrdU, EdU, or a TUNEL assay) and/or pyknotic (DAPI) nuclei were calculated separately in each bin. Two different cortical slices were systematically analyzed per animal. The number of animals used in each experiment is indicated in the figure legends.

Statistical analyses were conducted with Graphpad Prism (La Jolla, CA, Version 5.0c) using two-way analysis of variance and Bonferroni multiple comparison post hoc tests or Mann–Whitney *U* test, with significance measured at *, *p* < .05; **, *p* < .01; or ***, *p* < .001.

## RESULTS

### Radiation-Induced Apoptosis of Radial Glial Cells and Intermediate Progenitors

**Figure 1:**
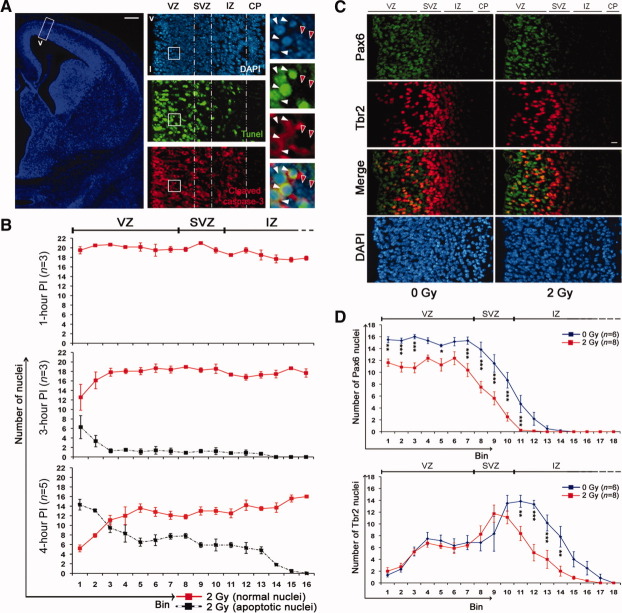
Radiation-induced apoptosis in the developing cortex. **(A):** Left: Coronal section of the cerebral hemisphere of an E14.5 embryo stained with DAPI. The white box defines the standard region in which analyses were performed. Scale bar = 200 μm. Middle: DAPI (blue), TUNEL (green), and cleaved caspase-3 (red) staining at 4 hours PI; scale bar = 10 μm. Right: Enlarged and merged images of the staining patterns within the white boxed areas. White arrows, apoptotic nuclei; red arrows, normal nuclei. **(B):** Number of normal (red) and apoptotic (pyknotic, black) nuclei per bin at 1, 3, and 4 hours PI. Mean values ± SEM were calculated from the indicated number (*n*) of embryos from at least three distinct litters. **(C):** Pax6 (green), Tbr2 (red), and DAPI (blue) staining. Scale bar = 10 μm. **(D):** Number of Pax6(+) nuclei per bin and Tbr2(+) nuclei per bin in nonirradiated (blue) and irradiated (red) embryos at 4 hours PI. Mean values ± SEM were calculated from the indicated number (*n*) of embryos from at least three litters. Statistical analysis was performed using Bonferroni post hoc tests. Abbreviations: CP, cortical plate; DAPI, 4′-6-diamidino-2-phenylindole; IZ, intermediate zone; PI, post-irradiation; SVZ, subventricular zone; TUNEL, terminal deoxynucleotidyl transferase labeling; V, ventricle; VZ, ventricular zone.

By TUNEL assay, the detection of pyknotic nuclei and the immunodetection of cleaved caspase-3, we found that in utero 2 Gy irradiation induces a high level of apoptosis in the cortical proliferative zone but not in the CP of mouse embryos at E14.5 (Fig. 1A). Since these experiments produced similar results, we further determined the kinetics of radiation-induced apoptosis by quantifying the number of pyknotic nuclei in a standard coronal sector of the dorsomedial cerebral wall at different time points after irradiation (Fig. 1B). This sector was 100 μm in its medial-lateral dimension and was divided into 18 bins of 10-μm height in its radial dimension. The sector was aligned such that the first bin was at the ventricle surface with its long axis parallel to the ventricle border, as previously described [20] (Supporting Information Fig. S1A).

The first apoptotic nuclei appeared at 3 hours PI in the two bins near the ventricle (Fig. 1B). At 4 hours PI, the number of apoptotic nuclei per bin was maximal near the ventricle and decreased progressively from the ventricular margin to the IZ (Fig. 1B). Rare apoptotic cells were also detectable in the CP (Fig. 1A). Radiation induced a significant decrease in the number of cells expressing Pax6, a marker of radial glial cells, and/or Tbr2, a marker of intermediate progenitors, and early postmitotic neurons [21] (Fig. 1C, 1D).

### Most Cells Irradiated in G2/M Die Within 4 Hours PI

**Figure 2:**
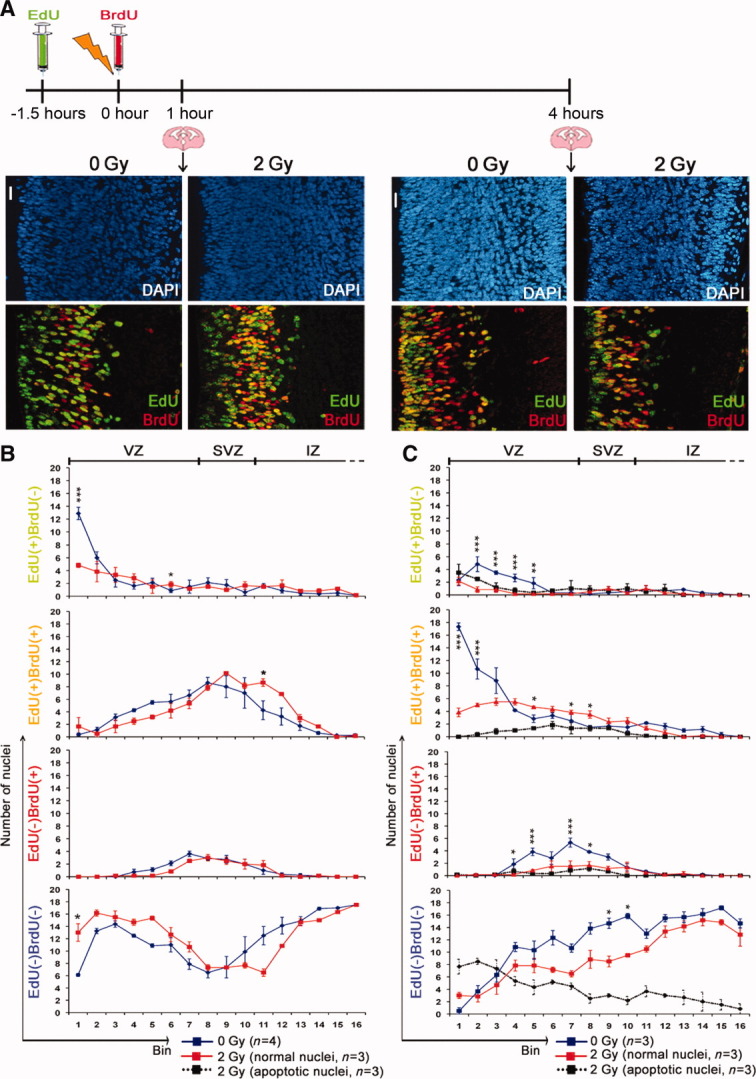
Cell cycle progression of irradiated neural stem and progenitor cell. **(A):** Schematic diagram of the experimental design and DAPI (blue), EdU (green), and BrdU (red) staining patterns found in coronal cortical slices at 1 and 4 hours PI (2 Gy). Scale bar = 20 μm. **(B, C):** Numbers/bin of EdU(+)BrdU(−), EdU(+)BrdU(+), EdU(−)BrdU(+), or EdU(−)BrdU(−) nuclei with a normal morphology in nonirradiated controls (blue) and with a normal (red) or apoptotic (pyknotic, black) morphology in irradiated (2 Gy) cortices at 1 hour (B) or 4 hours PI (C). No apoptotic nuclei were detected at 1 hour PI (B). Statistical analysis was performed using Bonferroni post hoc tests. Abbreviations: BrdU, 5-bromo-2′-deoxyuridine; DAPI, 4′-6-diamidino-2-phenylindole; EdU, 5-ethynyl-2′deoxyuridine; IZ, intermediate zone; SVZ, subventricular zone; VZ, ventricular zone.

To investigate the activation of checkpoints in irradiated NSPC, we performed pulse labeling of S-phase cells by using two distinct previously described analogs of thymidine, EdU and BrdU [22]. Pregnant mice were injected intraperitoneally with EdU at 1.5 hours before irradiation and with BrdU just after irradiation. We then determined the number and distribution of EdU(+) and BrdU(+) nuclei in cortical slices at 1 and 4 hours PI (Fig. 2). Importantly, neither EdU nor BrdU incorporation changed the level of radiation-induced apoptosis (Supporting Information Fig. S1B).

**Figure 3:**
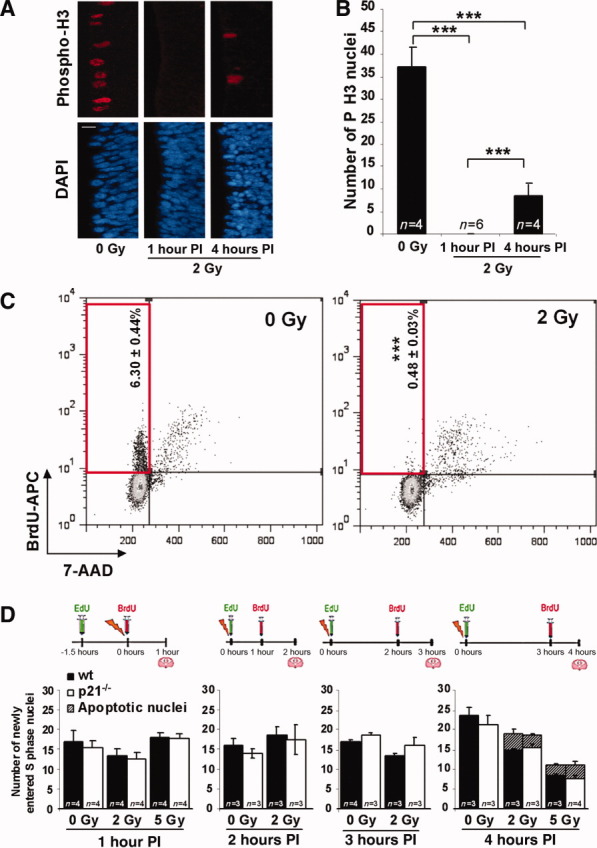
Induction of G2/M and intra-S checkpoints, but not a G1/S checkpoint, in irradiated radial glia within 4 hours PI.**(A):** PH3 staining at 1 and 4 hours PI (0 and 2 Gy). Scale bar = 10 μm. **(B):** Quantification of PH3(+) cells at the ventricular surface at 1 and 4 hours PI. Mean values ± SEM were calculated from the indicated number (*n*) of embryos from at least three distinct litters. Statistical analysis was performed using the Mann–Whitney *U* test. **(C):** FACS analyses of BrdU(+) cells from a microdissected E14.5 cortex at 4 hours PI (0 or 2 Gy). BrdU was injected at 0 hours PI. Representative cellular scales for BrdU and 7-AAD are given in arbitrary units. Red boxes indicate BrdU(+) G1 cells. Statistical analysis was performed using the Mann–Whitney *U* test. **(D):** Quantification of cells that entered S-phase during 1 to 4 hours PI (0, 2 or 5 Gy) in *wt* and *p21^−/−^* mice in different experiments, as represented schematically above each histogram. The mean values ± SEM were calculated from the indicated number (*n*) of embryos from at least two litters. Abbreviations: APC, allophycocyanin; 7-AAD, 7-amino-actinomycin D; BrdU, 5-bromo-2′-deoxyuridine; DAPI, 4′-6-diamidino-2-phenylindole; PI, post-irradiation; PH3, phospho-histone H3.

Our experimental design produced cells in G2 at 0 hours PI with EdU(+)BrdU(−) nuclei. The G2 and M phases of NSPCs have been reported to require 2 hours in the developing mouse brain [20]. Consistent with the basal to apical INM of radial glia after S-phase, most of the EdU(+)BrdU(−) nuclei in our analyses were found at 1 hour PI in the first bin near the ventricle in control brains (Fig. 2B), where they formed typical mitotic figures (Supporting Information Fig. S2A). Irradiation dramatically decreased the number of EdU(+)BrdU(−) nuclei at the ventricular surface (Fig. 2B) and induced the loss of nuclei expressing phospho-histone H3 (PH3), a marker of mitotic cells [23] (Fig. 3A, 3B). At 4 hours PI, these EdU(+)BrdU(−) nuclei were mainly apoptotic and still lower in number compared with the nonirradiated controls (Fig. 2C). However, most apoptotic nuclei present in the two bins near the ventricle in irradiated brains were not labeled by EdU and BrdU at 4 hours PI (Fig. 2C). Both their localization and the absence of EdU or BrdU labeling strongly suggested that these nuclei were irradiated in either late G2 or M phase, remained abnormally close to the ventricular surface as a consequence of a block or delay of INM after irradiation, and died thereafter.

Taken together, our data demonstrated that cells irradiated in either G2 or M phase are highly radiation sensitive and die within 4 hours PI after activation of the G2/M and mitotic checkpoints. Of note, mitoses reappeared at the ventricular surface of irradiated brains at 4 hours PI, as shown by the detection of PH3(+) nuclei (Fig. 3A, 3B), indicating that the G2/M checkpoint had already been passed in surviving cells.

### Induction of Intra-S-Phase Checkpoint

Injection of EdU 1.5 hours before irradiation and BrdU just after irradiation ensures that EdU(+)BrdU(+) nuclei correspond to cells irradiated in S-phase in cortical slices. Radial glial cells and intermediate progenitors enter S-phase between bins 4 and 10, thereafter referred to as S-phase bins. At 1 hour PI, EdU(+)BrdU(+) nuclei were detected in S-phase bins at similar numbers and distributions in irradiated and control brains (Fig. 2B), thus showing no obvious effects of radiation. Moreover, this result also shows that in our conditions BrdU detection allowed the detection of S-phase cells and not DNA synthesis associated with repair even after irradiation. At 4 hours PI, 19.0% ± 3.0% of EdU(+)BrdU(+) nuclei were apoptotic within S-phase bins indicating the activation of intra-S checkpoint in these cells. Fluorescence-activated cell sorting analysis revealed a nearly 13-fold decrease in the cortical NSPCs in S-phase at 0 hours PI that had achieved mitosis in irradiated brains at 4 hours PI (BrdU(+) cells with a 2*n* DNA content shown in Fig. 3C). Consistent with basal to apical INM in radial glial cells after S-phase completion, most EdU(+)BrdU(+) nuclei had migrated in the first three bins near the ventricles in unirradiated controls, 4 hours after BrdU injection (Fig. 2C). By contrast, in irradiated brains, few EdU(+)BrdU(+) nuclei had reached the ventricular surface (Fig. 2C) indicating that radiation also delayed INM in radial glial cells. These findings thus indicate that the activation of intra-S checkpoints in radial glia irradiated in S-phase delays INM and results in a significant level of cell death within 4 hours PI.

### Continuous S-Phase Entry of NSPCs Within 4 Hours PI

Injections of EdU at 1.5 hours before irradiation and BrdU just after irradiation enabled the identification of cells entering S-phase after irradiation via the detection of their EdU(−)BrdU(+) nucleus. At 1 hour PI, the number and localization within S-phase bins of EdU(−)BrdU(+) nuclei in irradiated brains remained similar to controls (Fig. 2B). The diffuse BrdU staining of these nuclei, typical of cells in early S-phase (Supporting Information Fig. S3A), confirmed the lack of a detectable block of S-phase entry within 1 hour PI. At 4 hours PI (Fig. 2C), 30.5% ± 4.7% of EdU(−)BrdU(+) nuclei were found to be apoptotic, indicating that these cells irradiated in G1 died during S-phase. In addition, their total numbers were markedly decreased in irradiated brains.

Since complete degradation of apoptotic nuclei could have contributed to this decrease, further experiments were performed in which EdU was injected just after irradiation. BrdU was administered at 1, 2, or 3 hours PI, and the animals were sacrificed 1 hour after BrdU injection (Fig. 3D). EdU(−)BrdU(+) nuclei were only detected within S-phase bins in irradiated brains (Supporting Information Fig. S3B), showing once again that the detection of BrdU incorporation after irradiation was not related to DNA synthesis associated with DNA repair. In these experiments, detection of EdU(−)BrdU(+) nuclei thus allowed the quantification of cells entering S-phase during the 2nd, 3rd, or 4th hour after irradiation, respectively. The results (Fig. 3D) clearly showed that 2 Gy irradiation did not reduce the S-phase entry from 1 to 4 hours PI, suggesting the lack of a block at the G1/S transition within 4 hours PI, contrary to the obvious activation of the other checkpoints within the same period of time, as shown above.

### Induction of p21-Dependent Apoptosis but a Lack of a p21-Mediated G1/S Checkpoint Within 4 Hours PI

**Figure 4:**
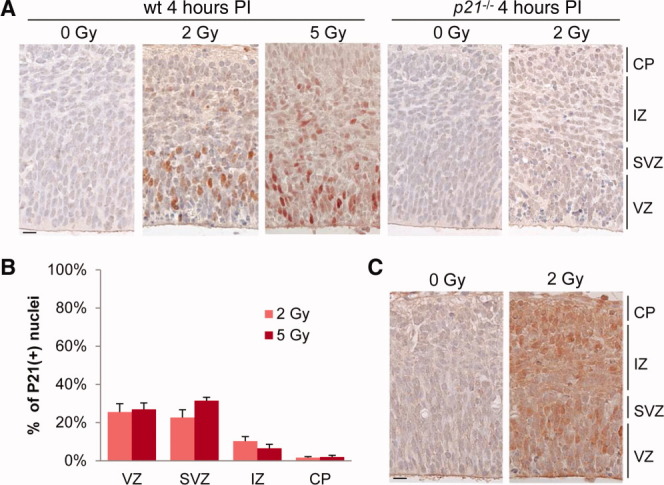
p21 expression is not induced in all irradiated cells. **(A):** Detection of p21 in coronal sections of E14.5 *wt* and *p21^−/−^* mice at 1 and 4 hours PI (0, 2, and 5 Gy). Scale bar = 10 μm. **(B):** Quantification of the number of p21-positive nuclei in the VZ, SVZ, IZ, and CP 4 hours after 2 or 5 Gy irradiation. **(C):** Detection of p53 in coronal sections of E14.5 *wt* mice at 4 hours PI (0 and 2 Gy). Scale bar = 10 μm. Abbreviations: CP, cortical plate; IZ, intermediate zone; PI, post-irradiation; SVZ, subventricular zone; VZ, ventricular zone.

p21 has been shown to play a crucial role in the radiation response at the G1/S checkpoint [1, 2]. We and others have previously reported that in contrast to our in vivo results, ionizing radiation induces a p21-dependent G1/S block in cultured mouse NSPCs in a p53-dependent manner [24, 25]. Immunohistochemistry in our current experiments revealed very low levels of p21 in nonirradiated brains, which increased in irradiated brains (Fig. 4A). Surprisingly, p21 was detected after 2 Gy irradiation in only around 20% of VZ and SVZ nuclei, 10% of IZ nuclei, and very rare in CP nuclei (Fig. 4B), whereas p53 was induced in almost all irradiated nuclei (Fig. 4C). Similar results were obtained at high-dose exposures (5 Gy, Fig. 4B).

**Figure 5:**
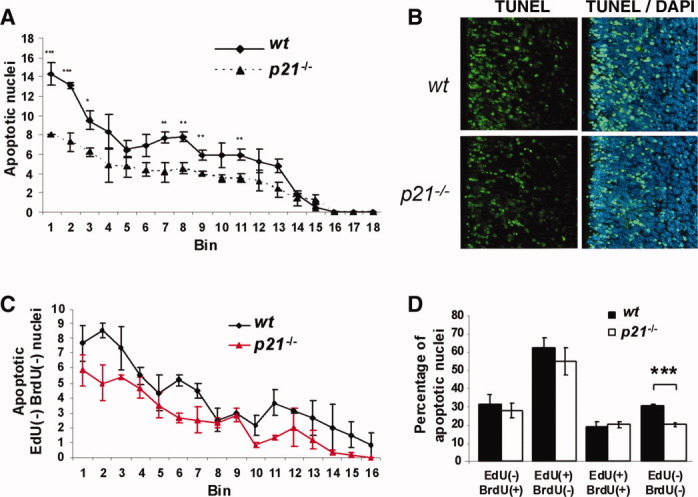
p21-dependent apoptosis at 4 hours PI. **(A):** Number of apoptotic nuclei/bin in *wt* and *p21^−/−^* mice at 4 hours PI. Mean values ± SEM were calculated from five embryos from at least three distinct litters. Statistical analysis was performed using Bonferroni post hoc tests. **(B):** TUNEL (green) and DAPI (blue) staining of cortical slices from irradiated *wt* and *p21^−/−^* mice at 4 hours PI. **(C):** Number of EdU(−)BrdU(−) apoptotic nuclei/bin in irradiated (2 Gy) *wt* and *p21^−/−^* mice cortices at 4 hours PI. Pregnant mice were injected with EdU at 1.5 hours before irradiation and with BrdU just after irradiation. **(D):** Comparison of the percentages of apoptotic EdU(−)BrdU(+), EdU(+)BrdU(−), EdU(+)BrdU(+), or EdU(−)BrdU(−) nuclei in the first 18 bins of cortical slices from irradiated *wt* and *p21^−/−^* mice at 4 hours PI. Pregnant mice were injected with EdU 1.5 hours before irradiation and with BrdU just after irradiation. Statistical analysis was performed using the Mann–Whitney *U* test. Abbreviations: BrdU, 5-bromo-2′-deoxyuridine; DAPI, 4′-6-diamidino-2-phenylindole; EdU, 5-ethynyl-2′deoxyuridine; TUNEL, terminal deoxynucleotidyl transferase labeling.

To determine the role of p21 in the DDR of NSPCs, we reproduced S-phase pulse labeling experiments with EdU and BrdU with *p21^−/−^* mice. No significant difference in the pattern of EdU and BrdU staining was observed between nonirradiated *p21^−/−^* and *wt* embryos, indicating that the p21 deficiency did not significantly alter the cell cycle progression of NSPCs in the developing brain (Supporting Information Fig. S4A). Moreover, we did not find any relevant differences between *wt* and *p21^−/−^* mice in terms of radiation-induced intra-S and G2/M checkpoints. Indeed, we found similar delays in INM and apoptosis induction in cells irradiated in S-phase (Supporting Information Fig. S4B, Fig. 2) and a similar block in mitosis entry of cells irradiated in G2 (Supporting Information Fig. S5). In contrast, we found a significant decrease in apoptosis in irradiated *p21^−/−^* mice at 4 hours PI suggesting a possible role of p21 in the radiation-induced apoptosis of neural cells (Fig. 5A, 5B). Injections of EdU 1.5 hour before irradiation and of BrdU just after irradiation showed that this was mainly the consequence of a significant decrease in apoptosis among EdU(−)BrdU(−) nuclei from the VZ to the IZ (Fig. 5C, 5D). With the exception of cells present at the border of ventricles that were likely to be in late G2/M at 0 hours PI, as discussed above, most of the EdU(−)BrdU(−) cells could correspond to expanding radial glial cells and intermediate progenitors irradiated in G1 or to differentiating cells migrating to the CP irradiated after their last mitosis. Importantly, this decrease in apoptosis had no influence on S-phase entry in irradiated *p21^−/−^* mice from 1 to 4 hours PI. Similar rates of cells entering S-phase and similar levels of cell death during S-phase were found in the two types of mice after irradiation (Fig. 3D).

We have previously described [19] the induction of similar numbers of γH2AX foci—a marker of double-strand breaks—/nuclei in the different zones of the 2 Gy-irradiated cortex at 1 hour PI. Quantification of γH2AX foci showed some cell-to-cell variations; however, all irradiated VZ cells exhibited DNA damages (Supporting Information Fig. S6). Therefore, to determine whether the lack of G1/S checkpoint was due to cell-to-cell variations in DNA damage levels, we tested the effects of other doses on S-phase entry after irradiation. As expected, lower doses (0.5 and 1 Gy) did not induce a decrease in S-phase entry (Supporting Information Fig. S7), despite the fact that quantifications of γH2AX foci revealed also the induction of DNA damages in all 1 Gy-irradiated VZ cells (Supporting Information Fig. S6). We have then tested the effects of 5 Gy to determine whether the lack of G1/S checkpoint was due to insufficient DNA damage levels—contrary to 2 Gy, 5 Gy is lethal just after birth for mouse embryos. As shown in Figure 3D, 5 Gy irradiation did not reduce the rate of S-phase entry during the 1st hour PI, confirming the lack of an immediate G1/S checkpoint in NSPCs. It significantly reduced the number of detected *wt* nuclei entering S-phase during the 4th hour PI, but the same decrease was observed in 5 Gy-irradiated *p21^−/−^* mice, indicating that this phenomenon was not related to the p21-dependent G1/S checkpoint.

Taken together, our current data reveal the lack of a detectable p21-dependent G1/S arrest within 4 hours PI. They further demonstrate that p21-dependent apoptosis of EdU(−)BrdU(−) cells had no consequences for S-phase entry within 4 hours PI. Although important variations have been reported in function of the nature and fate of the NSPC [26], the mean duration of the G1 phase in NSPCs from E14 to E15 in the developing mouse brain ranges from 9 to 11 hours [20]. Our results thus show that p21-dependent apoptosis is not a response mechanism of radial glial cells and intermediate progenitors irradiated in mid/late G1.

### p21 Has No Influence on the Reconstitution of the VZ at 24 Hours PI

**Figure 6:**
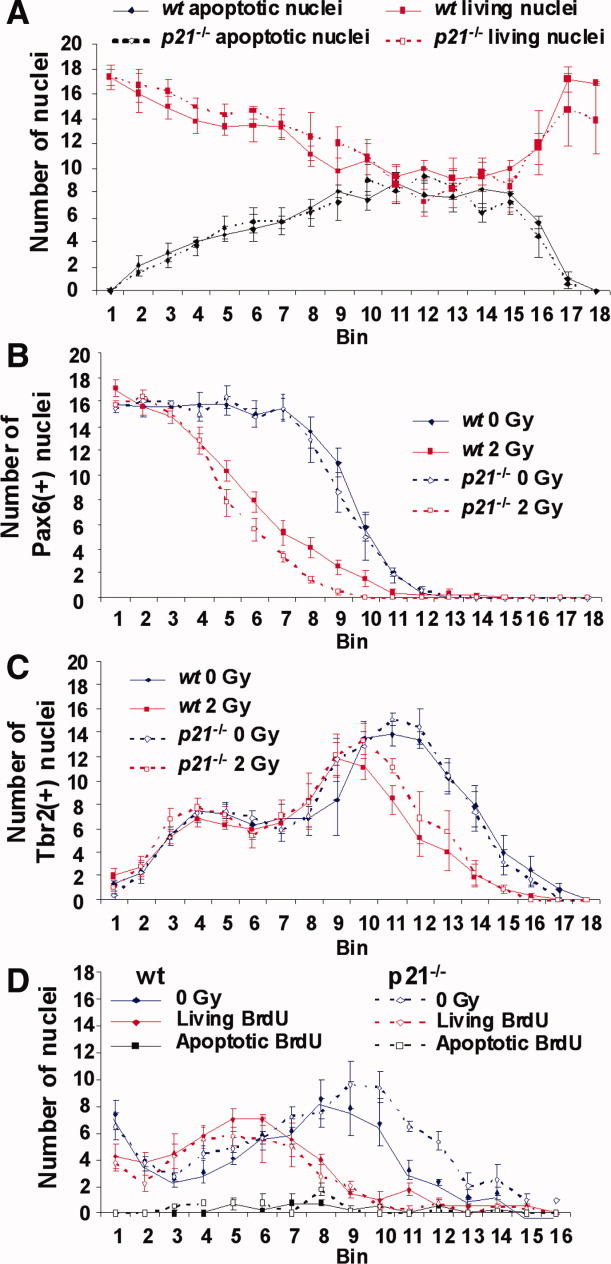
Restoration of the pool of proliferating radial glia at 24 hours PI. **(A):** Number of normal (red) and apoptotic (pyknotic, black) nuclei/bin in *wt* and *p21^−/−^* mice 24 hours after exposure to 2 Gy of radiation. Mean values ± SEM were calculated from six embryos from at least three distinct litters. **(B):** Number of Pax6(+) nuclei/bin in control (blue) and 2 Gy-irradiated (red) *wt* and *p21^−/−^* mice. Mean values ± SEM were calculated from four embryos from at least three litters. **(C):** Number of Tbr2(+) nuclei/bin in control (blue) and 2 Gy-irradiated (red) *wt* and *p21^−/−^* mice at 24 hours PI. Mean values ± SEM were calculated from three embryos from at least three litters. **(D):** Number of BrdU(+) nuclei/bin with a normal morphology in control mice (blue) and with a normal (red) or apoptotic (pyknotic, black) morphology in irradiated *wt* and *p21^−/−^* (right) mice at 24 hours PI. Pregnant mice were injected with BrdU at 2 hours before sacrifice. The mean values ± SEM were calculated from four embryos from at least three litters. Abbreviation: BrdU, 5-bromo-2′-deoxyuridine.

Recent in vitro studies have shown that the G1/S checkpoint may be slowly activated to fully block the S entry [27]. To determine whether p21-dependent G1/S checkpoint was activated at a later time point after irradiation in NSPCs, we compared *p21^−/−^* and *wt* mice at 24 hours PI. In both types of mice, living nuclei reappeared in the bins near the ventricle and the level of apoptosis reached a peak in overlying nuclear layers including the IZ, but not the CP, in which only rare apoptotic nuclei were detectable (Fig. 6A). Pax6 and Tbr2 immunostaining analysis produced strictly similar results in irradiated *p21^−/−^* and *wt* mice (Fig. 6B, 6C). Moreover, BrdU injection at 2 hours (22 hours PI, Fig. 6D) before sacrifice revealed the restoration of a pool of cycling NSPCs, for which the nuclei migrated to the ventricular surface (BrdU(+) nuclei) after S-phase completion in irradiated *wt* as well as *p21^−/−^* mice. The low levels of apoptotic BrdU(+) nuclei showed that radiation-induced apoptosis at 24 hours PI was not associated with intra-S checkpoints (Fig. 6D). Similarly, G2/M and mitotic checkpoints were no more activated by radiation, as shown by the similar percentages of mitotic cells near to the ventricle in both irradiated and control brains (Supporting Information Fig. S6) and the lack of apoptotic cells near the ventricle (Fig. 6A). These data collectively show that the p21 status has no influence on the effects of irradiation in the pool of NSPCs at 24 hours PI and rules out the induction of a p21-dependent G1-checkpoint by this stress stimulus within 4 and 24 hours PI.

**Figure 7:**
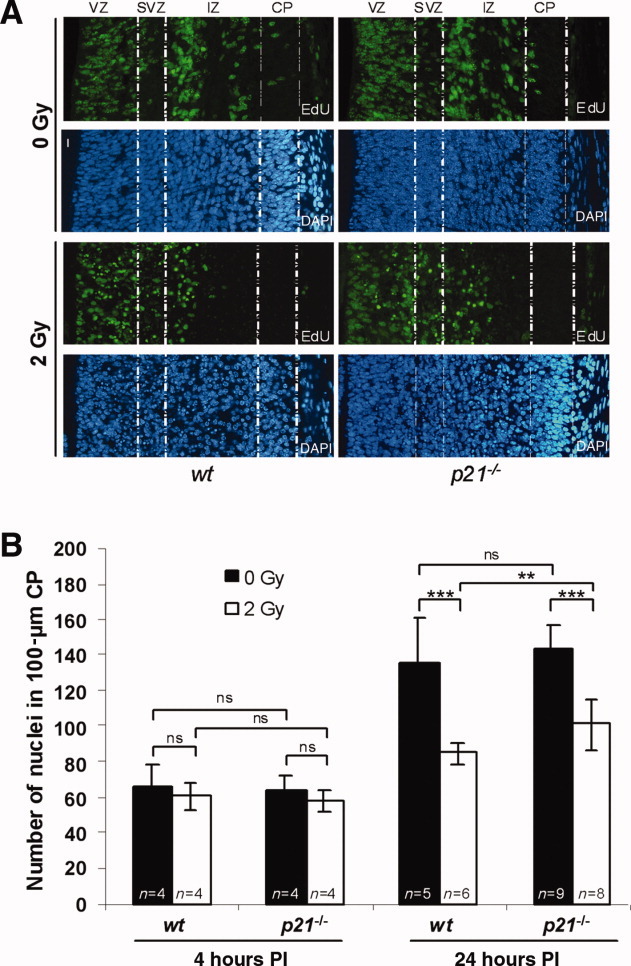
The arrival of postmitotic neurons in the CP within 24 hours PI is decreased to a lesser extent in *p21^−/−^* than in *wt* mice. **(A):** Representative fluorescence micrographs of DAPI (blue) and EdU (green) staining of nonirradiated and 2 Gy-irradiated cortical slices of embryonic *wt* and *p21*^−/−^ mice at 24 hours PI (EdU was injected just after irradiation). Scale bar = 10 μm. **(B):** Quantification of the number of nuclei in the CP of *wt* and *p21^−/−^* mice within a standard sector of a 100-μm width at 4 and 24 hours PI (0 and 2 Gy). The mean values ± SEM were calculated from the indicated number (*n*) of embryos from at least three litters. Statistical analysis was performed using the Mann–Whitney *U* test. Abbreviations: CP, cortical plate; DAPI, 4′-6-diamidino-2-phenylindole; EdU, 5-ethynyl-2′deoxyuridine; IZ, intermediate zone; PI, post-irradiation; SVZ, subventricular zone; VZ, ventricular zone.

Another important conclusion of our current results is that the cells that survived at 4 hours PI in *p21^−/−^* mice had no impact on the composition of the VZ at 24 hours PI, strongly suggesting that the apoptotic effects of p21 were in cells irradiated after their last mitosis. To confirm this possibility, we studied the impact of p21 deficiency on the arrival of new cells in the CP after irradiation. In the experiment, we quantified the nuclei within the CP in a standard sector of 100-μm width at 4 and 24 hours PI (Fig. 7A, 7B). The number of nuclei was twofold increased from 4 to 24 hours in both nonirradiated *p21^−/−^* mice and *wt* controls. Injection of EdU at 0 hours PI revealed that this increase was due essentially to cells that had completed their last S-phase before 0 hours PI, since we found very low number of EdU(+) nuclei in the CP (Fig. 7A). Radiation dramatically reduced the arrival of cells in the CP within the same time frame, but to a lesser extent in *p21^−/−^* compared with *wt* mice (Fig. 7B). Hence, these data are consistent with the contention that there is a proapoptotic effect of p21 in postmitotic migrating cells at 4 hours PI.

## DISCUSSION

In this study, we show for the first time that DDR in NSPCs in the developing cortex differs from the classic model. These cells activate S and G2/M checkpoints but unexpectedly do not initiate a detectable G1/S block, indicating that the G1/S checkpoint is not involved in the choice between repair or elimination of damaged cells. It has been previously reported that p53 plays a central role in the DDR in the developing brain, thus showing that this tumor suppressor is required for radiation-induced apoptosis [17]. We found in our present experiments that p21, normally considered to be the principal mediator of p53-dependent cell cycle arrest in response to DNA damage, does not have such a role in irradiated radial glial cells, the cells with neural stem cell properties in the developing brain.

The length of the cell cycle has been shown to depend on the types of NSPC present in the developing brain. Indeed, G1 lengthening has been associated with the transition from stem radial glia to intermediate progenitors [26] and with a subclass of VZ cells directly producing postmitotic neurons [28]. Moreover, a recent paper has reported that expanding radial glia and intermediate progenitors exhibit a substantially longer S-phase than radial glial cells and intermediate progenitors committed to neuron production [29]. We have shown here that the lack of G1/S checkpoint concerned all these different types of NSPC.

A further finding of this study is that, in contrast to the neurons of the CP, migrating postmitotic cells are highly sensitive to radiation-induced apoptosis. We found from our analysis that p21 is involved in the apoptotic response in these cells at 4 hours PI but not at 24 hours PI. Although p21 has been reported to prevent cell death by inducing cell cycle blocks, providing time for the repair of damaged cells, many studies have also shown that it could have a proapoptotic role in some cell types [30–32]. Our results suggest that the induction of p21 after radiation exposure has deleterious effects on postmitotic migrating neurons, although the molecular mechanisms remained to be determined.

Although the level of p21 expression was found to be increased by ionizing radiation in some VZ cells in irradiated brain as anticipated, we showed that many living cells in the proliferating zone still expressed no or low levels of p21 after irradiation. This suggests that the lack of a G1/S arrest is related to the lack of or an insufficient radiation-induced expression of p21. It has been previously shown that p21 is strongly repressed by multiple transcription factors in neural cells during development such as FoxG1, Bmi1, Olig2, and RunX, which have been shown also to promote the amplification of NSPCs [9, 13–15]. These findings indicate that in contrast to p27, p21 is only expressed during the G1 to G0 transition in the developing brain [16]. Further experiments will be needed, however, to determine whether these transcription factors are also responsible for the lack of p21-mediated G1/S checkpoint in irradiated NSPCs by counteracting the radiation-induced p53-dependent signaling, which normally stimulates p21 expression.

We and others have previously reported that, in vitro, ionizing radiation induces both p21 expression and a G1/S block in neural progenitors cultured in neurospheres in a p53-dependent manner [24, 25]. Discrepancies with our current in vivo data probably result from the specific organization of the developing cortex and specific cell-to-cell interactions that are not reproduced in vitro. Notch signaling pathway represents a key regulator of NSPC. It has been recently shown that the inhibition of Notch signaling in human embryonic stem cell-derived neural stem cells delays the G1/S-phase transition and accelerates neuronal differentiation in vitro and in vivo [33]. Moreover, the G1 arrest induced by x-ray irradiation in NSPC cultures has been shown to be associated with the inactivation of Notch [25]. Further experiments are needed to determine whether activation of Notch signaling pathway, ensured by the specific organization of the developing cortex, is involved in the mechanisms responsible for the lack of induction of G1/S checkpoints in vivo in NSPCs after irradiation.

The length of the G1 phase has been shown to play a central role in influencing the switch of NSPCs from expansion to differentiation modes. The lengthening of G1 by inhibition of CDKs triggers premature neurogenesis [34], whereas CDK4/CyclinD1 overexpression in NSPC shortens G1, delays neurogenesis, and promotes the generation and expansion of intermediate progenitors [35]. If radiation had induced a block at the G1/S transition in NSPCs, it would have had similar consequences, enhancing its deleterious effects by reducing the pool of cycling radial glial cells. However, no increase in neurogenesis was observed in the irradiated mouse brains and we instead observed, concomitantly with a massive radiation-induced apoptotic response, the relatively rapid restoration of the pool of cycling progenitors. The lack of a G1/S checkpoint response in radial glia could thus be considered part of an efficient DDR that favors the reconstitution of the neural stem cell compartment, which could have been otherwise far more severely impaired.

This lack of a G1/S checkpoint is nevertheless intriguing since its loss is usually considered to be an important oncogenic step leading to augmented tumor cell proliferation in some malignancies, thereby contributing to mutagenesis and conferring a survival advantage. However, we have shown in our current data that S and G2/M checkpoints are major components in the DDR of radial glia, and that the lack of G1/S checkpoint could be compensated by the activation of an intra-S checkpoint in severely damaged cells that have entered S-phase after irradiation. Moreover, the high level of apoptosis at 24 hours PI in migrating postmitotic neurons generated from cells irradiated while cycling may also compensate for the lack of a G1/S checkpoint, reducing the risk of critically damaged cells continuing to survive.

## CONCLUSIONS

In conclusion, our current study shows that the DDR of radial glial cells does not involve a G1/S checkpoint in the developing brain, which is of great significance considering the current view of the importance of this checkpoint. These findings raise the question of whether this phenomenon extends to other types of stem cells.

## Supporting Information

Figure S1 The incorporation of EdU/BrdU does not affect the irradiation-induced apoptotic response (A) Fluorescent micrographs of DAPI (blue) stained cortical slices at 4 hPI from a standard sector of the dorsomedial cerebral wall. This sector was 100 μm in its medial-lateral dimension divided into 18 bins and was 10 μm in height in its radial dimension. The sector was aligned such that the first bin was at the ventricle (V) surface, with its long axis parallel to the ventricle border. Scale bar, 10 μm. (B) Comparison of numbers of apoptotic nuclei per bin in mice injected with EdU/BrdU (black) versus non-injected mice (red) at 4 hPI. Mean values ± SEM were calculated from five embryos from at least three distinct litters. No statistically significant differences (as assessed by Bonferroni post hoc tests) were found between the numbers of apoptotic nuclei in mice injected with EdU/BrdU and non-injected mice.

Figure S2 Induction of a G2/M but not a G1/S checkpoint in irradiated radial glia at 1 hPI Mice were injected with EdU (green) at 1.5 h before and with BrdU (red) just after irradiation. Cortical slices were then analyzed at 1 hPI. EdU(+)BrdU(−) mitotic nuclei (white arrows) were evident at the border of the ventricle (v) in non-irradiated mice but not found in irradiated brains at 1 hPI.

Figure S3 Detection of cells entering S phase after irradiation (A) Fluorescent micrographs of DAPI (grey), EdU (green) and BrdU (red) stained cortical slices at 1 hPI. White arrows highlight nuclei with diffuse BrdU staining, typical of cells in early S phase. Scale bar, 10 μm. (B) Numbers of EdU(−)BrdU(+) nuclei per bin in cortical slices from non-irradiated controls (blue) and with a normal (red) or apoptotic (pyknotic, black) morphology in cortical slices from irradiated (2Gy) wt mice at 1, 2, 3 and 4 hPI. Mean values ± SEM were calculated from 3 to 4 embryos from at least two distinct litters.

Figure S4 Cell cycle progression of p21 null neural progenitors at 1 and 4 hPI (2 Gy) (A) Top: Schematic diagram of the experimental design. Bottom: Numbers per bin of EdU(+)BrdU(−), EdU(+)BrdU(+), EdU(−)BrdU(+) or EdU(−)BrdU(−) nuclei in cortical slices from non-irradiated wt (blue) and p21 null (red) mice at 4 hPI. Statistically significant differences (as assessed by Bonferroni post hoc tests) between numbers of nuclei in p21 null and wt mice are indicated. Mean values ± SEM were calculated from six embryos from at least two distinct litters. (B) Top: Schematic diagram of experimental design. Bottom: Representative fluorescence micrographs of DAPI staining (blue), and of EdU (green) and BrdU (red) detection in coronal cortical slices. Scale bars, 20 μm. (C, D) Numbers per bin of EdU(+)BrdU(−), EdU(+)BrdU(+), EdU(−)BrdU(+) and EdU(−)BrdU(−) nuclei with a normal morphology in cortical slices from non-irradiated controls (blue) and with a normal (red) or apoptotic (pyknotic, black) morphology in cortical slices from irradiated (2 Gy) p21 null mice at 1 h (C) or 4 h (D) PI. No apoptotic nuclei were found at 1 hPI (C). Statistically significant differences (as assessed by Bonferroni post hoc tests) between the numbers of normal nuclei in irradiated and control p21 null mice are indicated.

Figure S5 Induction of S and G2/M checkpoints in irradiated neural progenitors of wt and p21 null mice Quantification of phospho-histone H3-positive cells along the cortical ventricular surface at 1, 4 and 24 hPI (2 Gy) in wt and p21 null mice. Mean values ± SEM were calculated from the indicated number of embryos from at least three distinct litters. Significant differences were assessed using the Mann-Whitney test.

Figure S6 Quantification of γH2AX foci in irradiated VZ nuclei (A) γH2AX (green) staining at 1h PI (0 and 2 Gy) and DAPI (gray) staining. Scale bar, 10 μm. (B) Distribution of the numbers of γH2AX foci per nuclei within the VZ at 1 h PI at 1 Gy (blue, n=151 nuclei analyzed) and 2 Gy (red, n=210 nuclei analyzed).

Figure S7 Absence of G1/S checkpoint, in irradiated radial glia even at lower doses Quantification of cells that entered S phase during 1 hPI (0 and 1 Gy) and 3 hPI (0, 0.5, or 1 Gy) in wt mice in different experiments, as represented schematically above each histogram. The mean values ± SEM were calculated from the indicated number (n) of embryos from at least two litters.
