# Supplementary figures and images for: Lack of a p21waf1/cip-Dependent G1/S Checkpoint in Neural Stem and Progenitor Cells After DNA Damage In Vivo
Source: Stem Cells. 2011 Dec 12;30(3):537–47. doi: 10.1002/stem.1010 (PMC3378718; doi:10.1002/stem.1010)

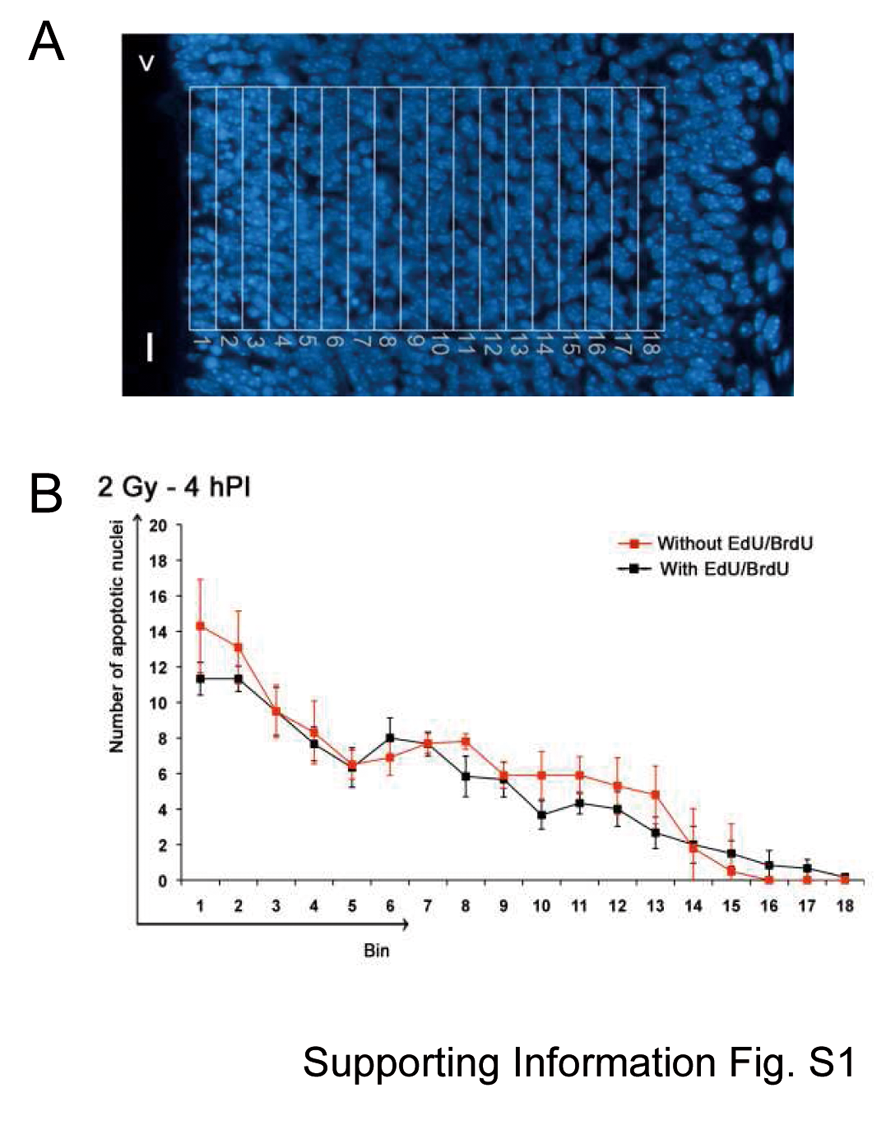

Supplement: Figure S1 — The incorporation of EdU/BrdU does not affect the irradiation-induced apoptotic response (A) Fluorescent micrographs of DAPI (blue) stained cortical slices at 4 hPI from a standard sector of the dorsomedial cerebral wall. This sector was 100 μm in its medial-lateral dimension divided into 18 bins and was 10 μm in height in its radial dimension. The sector was aligned such that the first bin was at the ventricle (V) surface, with its long axis parallel to the ventricle border. Scale bar, 10 μm. (B) Comparison of numbers of apoptotic nuclei per bin in mice injected with EdU/BrdU (black) versus non-injected mice (red) at 4 hPI. Mean values ± SEM were calculated from five embryos from at least three distinct litters. No statistically significant differences (as assessed by Bonferroni post hoc tests) were found between the numbers of apoptotic nuclei in mice injected with EdU/BrdU and non-injected mice. [file stem0030-0537-SD1.tif]

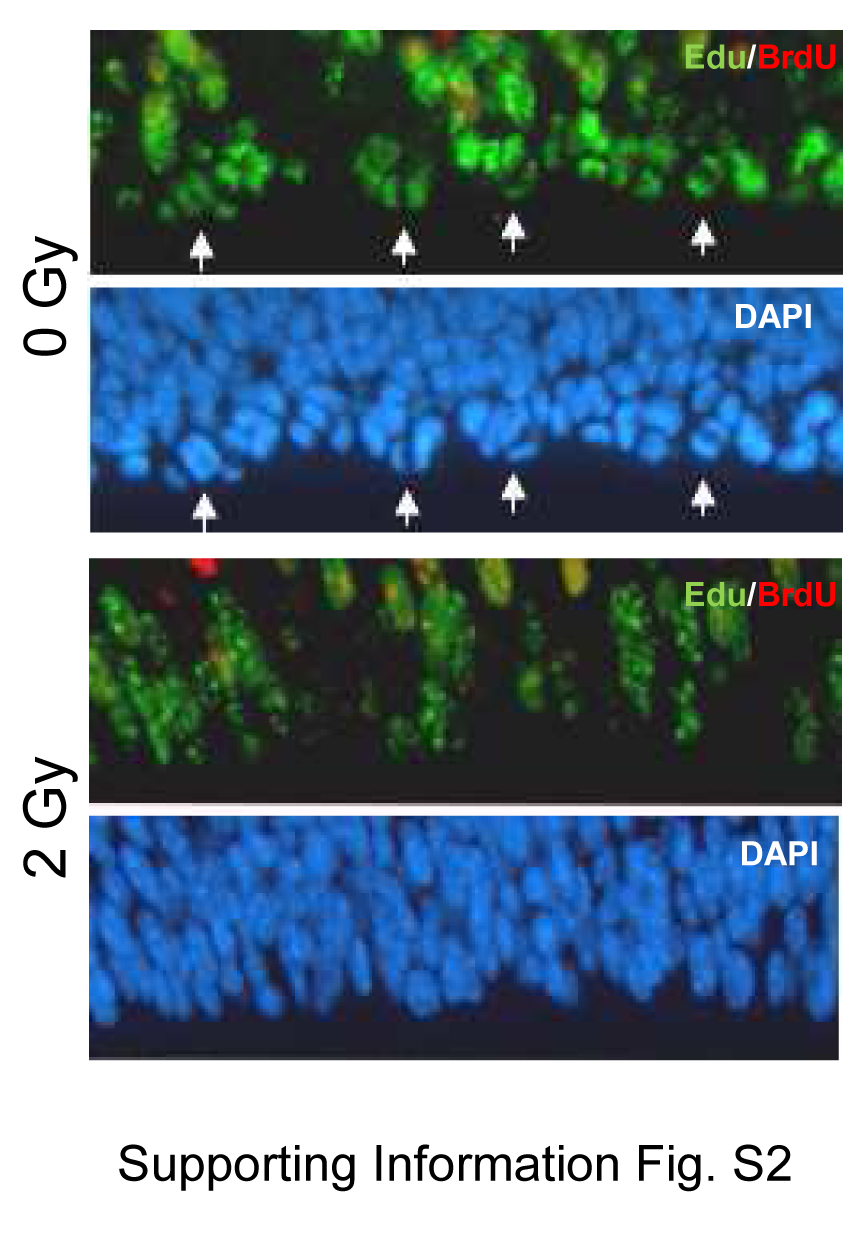

Supplement: Figure S2 — Induction of a G2/M but not a G1/S checkpoint in irradiated radial glia at 1 hPI Mice were injected with EdU (green) at 1.5 h before and with BrdU (red) just after irradiation. Cortical slices were then analyzed at 1 hPI. EdU(+)BrdU(−) mitotic nuclei (white arrows) were evident at the border of the ventricle (v) in non-irradiated mice but not found in irradiated brains at 1 hPI. [file stem0030-0537-SD2.tif]

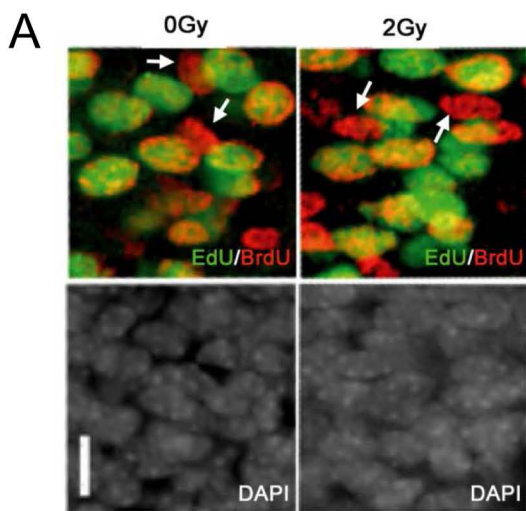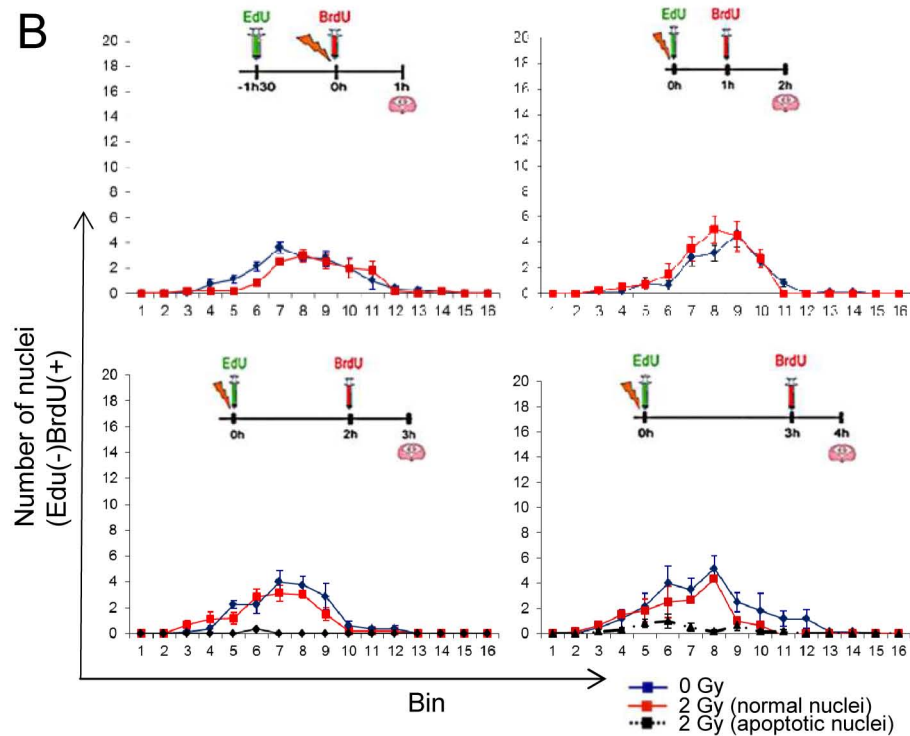

Supporting Information Fig. S3

Supplement: Figure S3 — Detection of cells entering S phase after irradiation (A) Fluorescent micrographs of DAPI (grey), EdU (green) and BrdU (red) stained cortical slices at 1 hPI. White arrows highlight nuclei with diffuse BrdU staining, typical of cells in early S phase. Scale bar, 10 μm. (B) Numbers of EdU(−)BrdU(+) nuclei per bin in cortical slices from non-irradiated controls (blue) and with a normal (red) or apoptotic (pyknotic, black) morphology in cortical slices from irradiated (2Gy) wt mice at 1, 2, 3 and 4 hPI. Mean values ± SEM were calculated from 3 to 4 embryos from at least two distinct litters. [file stem0030-0537-SD3.pdf]

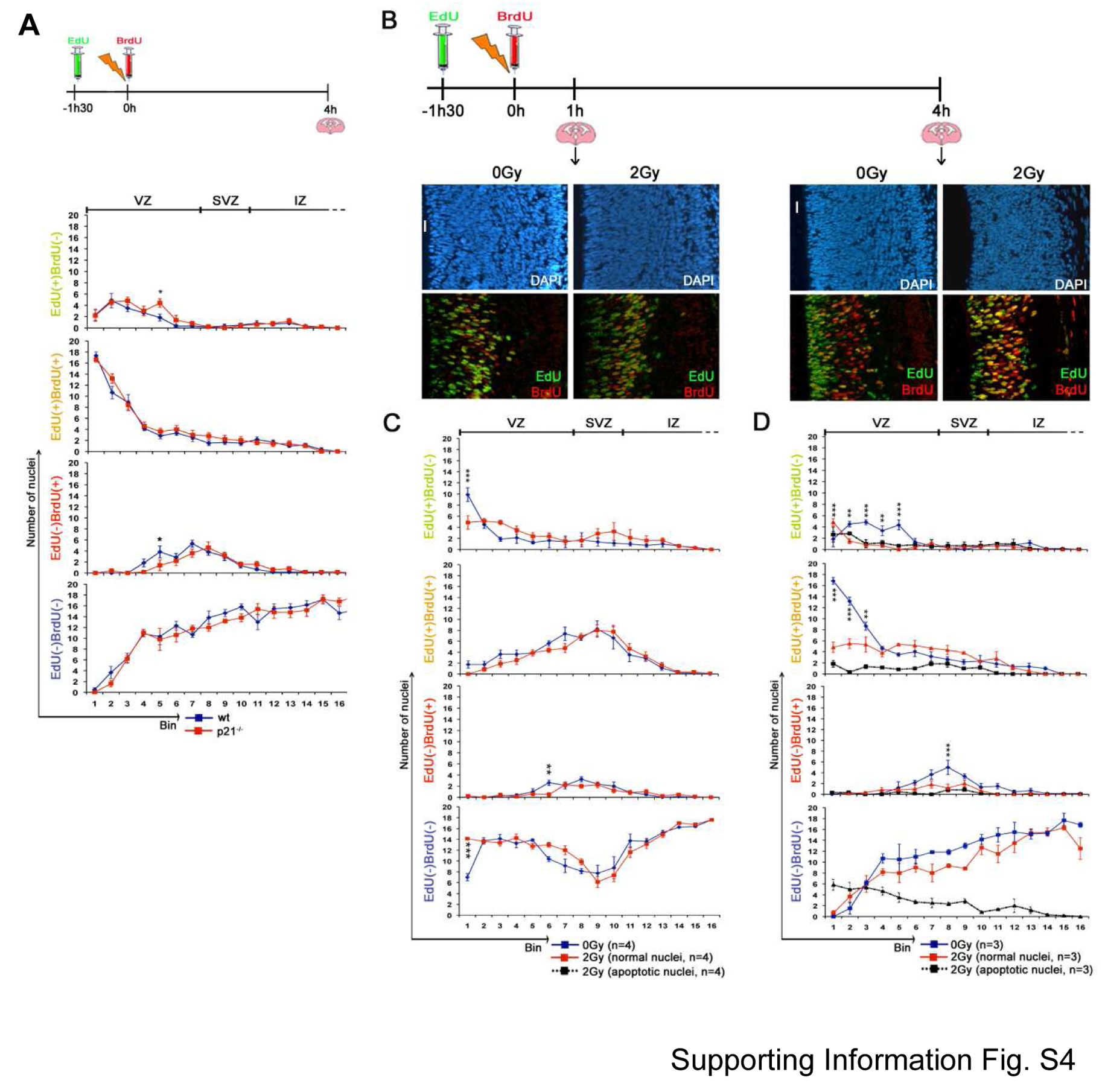

Supplement: Figure S4 — Cell cycle progression of p21 null neural progenitors at 1 and 4 hPI (2 Gy) (A) Top: Schematic diagram of the experimental design. Bottom: Numbers per bin of EdU(+)BrdU(−), EdU(+)BrdU(+), EdU(−)BrdU(+) or EdU(−)BrdU(−) nuclei in cortical slices from non-irradiated wt (blue) and p21 null (red) mice at 4 hPI. Statistically significant differences (as assessed by Bonferroni post hoc tests) between numbers of nuclei in p21 null and wt mice are indicated. Mean values ± SEM were calculated from six embryos from at least two distinct litters. (B) Top: Schematic diagram of experimental design. Bottom: Representative fluorescence micrographs of DAPI staining (blue), and of EdU (green) and BrdU (red) detection in coronal cortical slices. Scale bars, 20 μm. (C, D) Numbers per bin of EdU(+)BrdU(−), EdU(+)BrdU(+), EdU(−)BrdU(+) and EdU(−)BrdU(−) nuclei with a normal morphology in cortical slices from non-irradiated controls (blue) and with a normal (red) or apoptotic (pyknotic, black) morphology in cortical slices from irradiated (2 Gy) p21 null mice at 1 h (C) or 4 h (D) PI. No apoptotic nuclei were found at 1 hPI (C). Statistically significant differences (as assessed by Bonferroni post hoc tests) between the numbers of normal nuclei in irradiated and control p21 null mice are indicated. [file stem0030-0537-SD4.tif]

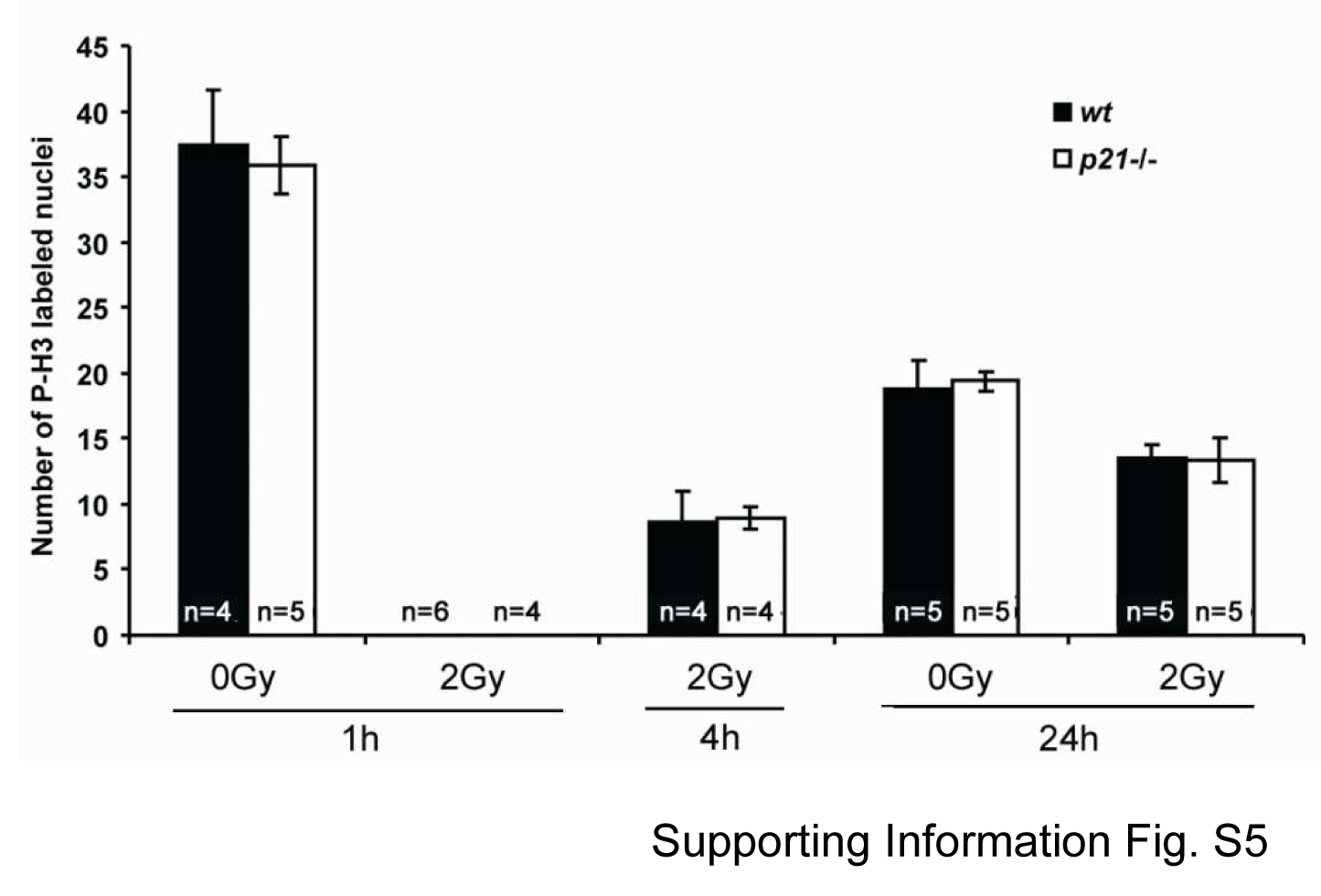

Supplement: Figure S5 — Induction of S and G2/M checkpoints in irradiated neural progenitors of wt and p21 null mice Quantification of phospho-histone H3-positive cells along the cortical ventricular surface at 1, 4 and 24 hPI (2 Gy) in wt and p21 null mice. Mean values ± SEM were calculated from the indicated number of embryos from at least three distinct litters. Significant differences were assessed using the Mann-Whitney test. [file stem0030-0537-SD5.tif]

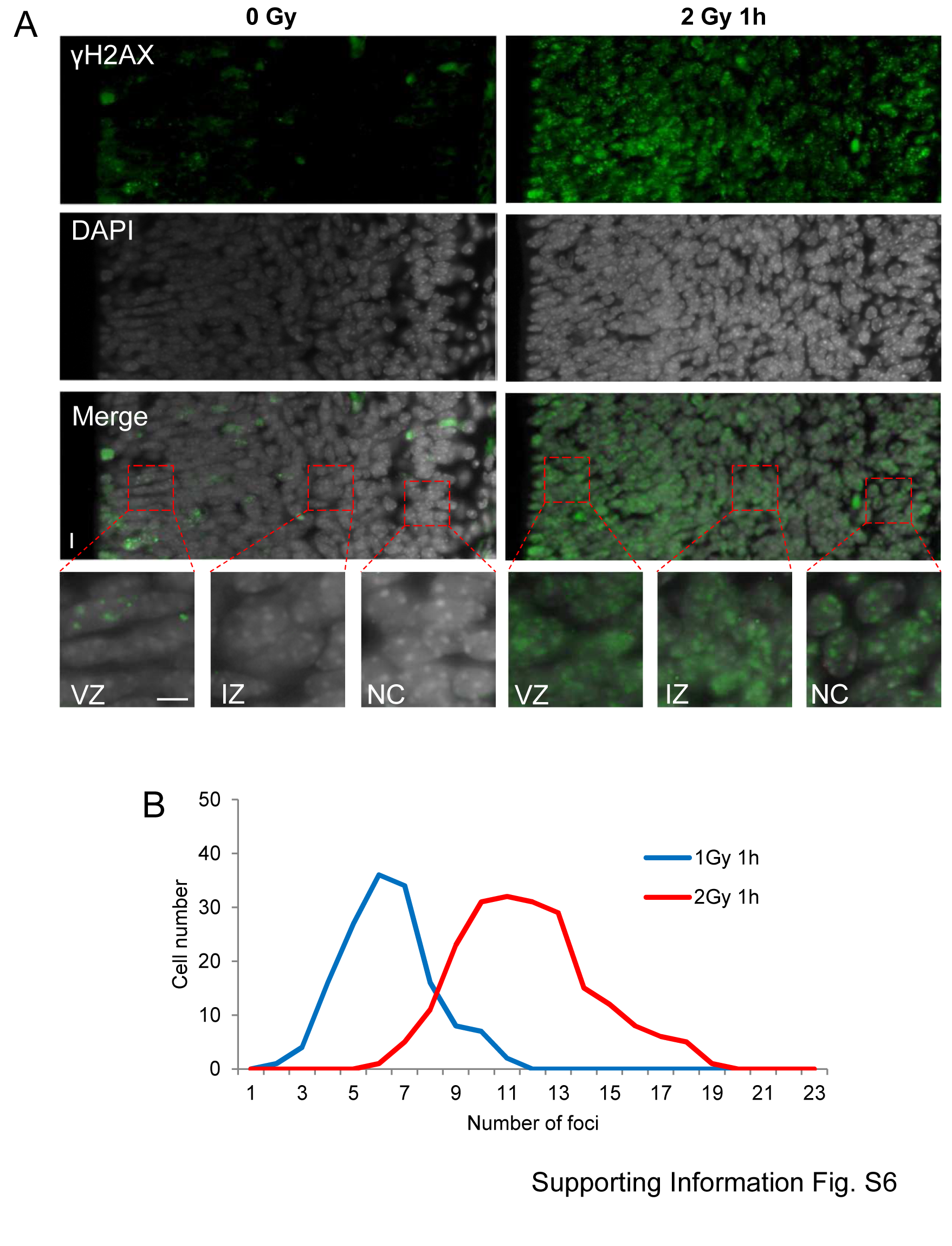

Supplement: Figure S6 — Quantification of γH2AX foci in irradiated VZ nuclei (A) γH2AX (green) staining at 1h PI (0 and 2 Gy) and DAPI (gray) staining. Scale bar, 10 μm. (B) Distribution of the numbers of γH2AX foci per nuclei within the VZ at 1 h PI at 1 Gy (blue, n=151 nuclei analyzed) and 2 Gy (red, n=210 nuclei analyzed). [file stem0030-0537-SD6.tif]

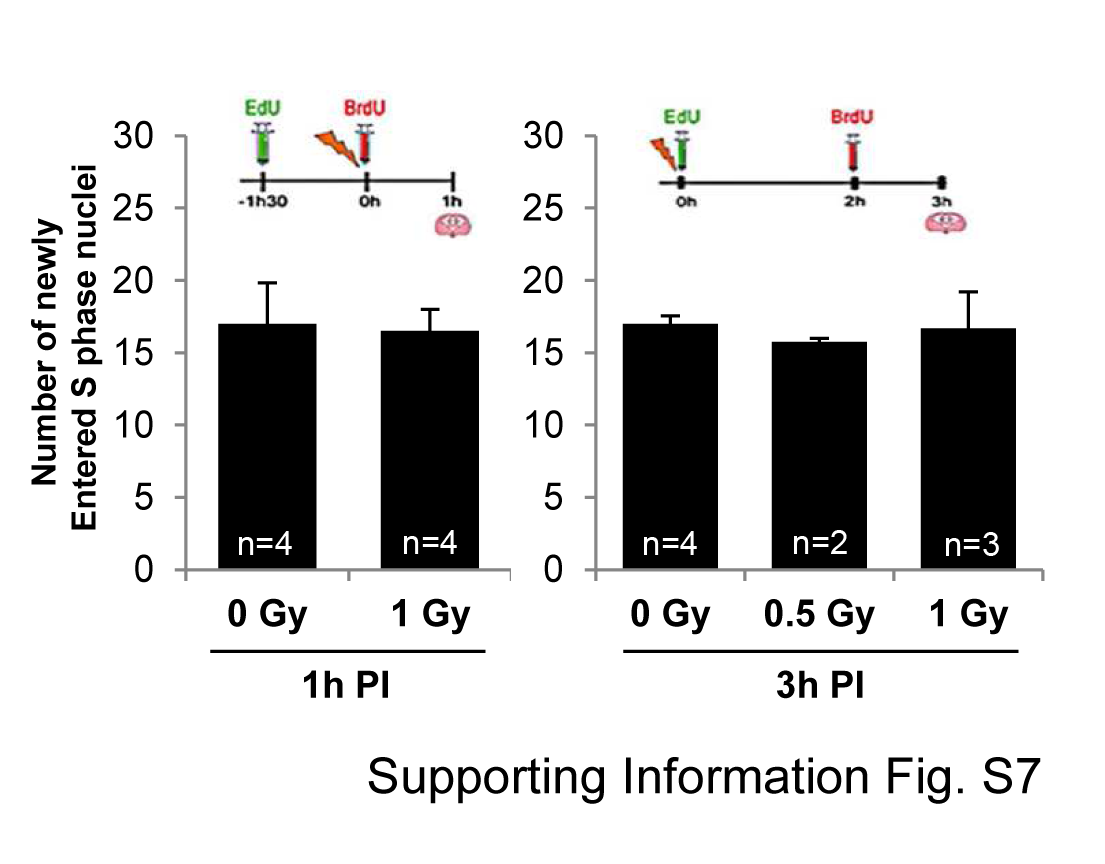

Supplement: Figure S7 — Absence of G1/S checkpoint, in irradiated radial glia even at lower doses Quantification of cells that entered S phase during 1 hPI (0 and 1 Gy) and 3 hPI (0, 0.5, or 1 Gy) in wt mice in different experiments, as represented schematically above each histogram. The mean values ± SEM were calculated from the indicated number (n) of embryos from at least two litters. [file stem0030-0537-SD7.tif]
